# Supplementary material for: Analysis of PPARγ Signaling Activity in Psoriasis
Source: Int J Mol Sci. 2021 Aug 10;22(16):8603. doi: 10.3390/ijms22168603 (PMC8395241; doi:10.3390/ijms22168603)
Supplement: Supplementary file 1 [file ijms-22-08603-s001.zip › Supplemental materials_Analysis of PPARg signaling activity in psoriasis/Pathway models/Models images and html files/Model of PPARG signaling in psoriasis/5237.html]

CXCR3


# Protein CXCR3

|  |  |
| --- | --- |
| URN | urn:agi-llid:2833 |
| Total Entities | 0 |
| Connectivity | 5816 |
| Name | CXCR3 |
| Description | chemokine (C-X-C motif) receptor 3 |
| Notes | This gene encodes a G protein-coupled receptor with selectivity for three chemokines, termed CXCL9/Mig (monokine induced by interferon-g), CXCL10/IP10 (interferon-g-inducible 10 kDa protein) and CXCL11/I-TAC (interferon-inducible T cell a-chemoattractant). Binding of chemokines to this protein induces cellular responses that are involved in leukocyte traffic, most notably integrin activation, cytoskeletal changes and chemotactic migration. Alternatively spliced transcript variants encoding different isoforms have been found for this gene. One of the isoforms (CXCR3-B) shows high affinity binding to chemokine, CXCL4/PF4 (PMID:12782716). [provided by RefSeq, Jun 2011] |
| Primary Cell Localization | Plasma membrane |
| Class | Receptor |

---

|  |  |
| --- | --- |
| Pathway | CXC Chemokine Receptor Signaling |
|  | Proplatelet Maturation |
|  | Find me Signal: Apoptotic Cell Attracts Phagocyte |
|  | Neutrophil Recruitment and Priming |
|  | Neutrophil Chemotaxis |
|  | Natural Killer Cell Precursor -> Natural Killer Cell Surface Expression Markers |
|  | CD8+ Naive T-cell -> CD4+ Naive T-cell Surface Expression Markers |
|  | Proteins Involved in Atherosclerosis |
|  | Chemokines Signaling in Atherosclerosis |
|  | Proteins Involved in Myocarditis |
|  | Proteins Involved in Melanoma |
|  | Hashimoto's Thyroiditis |
|  | Enterocyte Mediated Mucosal Damage in Celiac Disease |
|  | Celiac Disease |
|  | Th1-Cell Activation in Crohn's Disease |
|  | Leukocyte Migration toward the Endothelial Cell in Ulcerative Colitis |
|  | B-Cell Chronic Lymphocytic Leukemia |
|  | Osteoclast Activation in Multiple Myeloma |
|  | Mast-Cell Related Chemokines Signaling in Asthma |
|  | Proteins Involved in Rheumatoid Arthritis |
|  | Proteins Involved in Multiple Sclerosis |
|  | Proteins Involved in Spontaneous Abortion |
|  | Proteins Involved in Chronic Obstructive Pulmonary Disease |
|  | Proteins Involved in Glomerulonephritis |
|  | IL15 Expression Targets |
|  | CD Markers -> Expression Targets in Lymphoid System and Blood |
|  | Model of PPARG signaling in psoriasis |
|  | 3\_Leukocyte migration toward the endothelial cells in the intestine microvasculature |
|  | 4\_1\_Polymorphisms associated with inflammatory bowel diseases |
|  | 1\_CD4+ T cell and epithelial cell response in celiac disease |
|  | drug-target pairs direct regulation |

---

|  |  |
| --- | --- |
| MedScan ID | 2833 |

---

|  |  |
| --- | --- |
| LocusLink ID | 2833 |
|  | 12766 |
|  | 84475 |

---

|  |  |
| --- | --- |
| Alias | chemokine receptor CXCR3 |
|  | chemokine (C-X-C) receptor III |
|  | IFN-inducible protein 10 receptor |
|  | IP-10/mig receptor |
|  | interferon-inducible protein 10 receptor |
|  | CD183 antigen |
|  | HuMig receptor |
|  | Mig-R |
|  | C-X-C chemokine receptor type 3 |
|  | Mig receptor |
|  | CXCR3 receptors |
|  | CXCR3 receptor |
|  | C-X-C chemokine receptor type III |
|  | chemokine receptors CXCR3 |
|  | GPR9 |
|  | MigR |
|  | interferon-gamma-inducible protein-10 receptor |
|  | IFN-gamma-inducible protein 10 receptor |
|  | CKR-L2 |
|  | CD183 |
|  | CCR3\_HUMAN |
|  | CMKAR3 |
|  | IFNG-inducible protein 10 receptor |
|  | CXC-R3 |
|  | IP-10/CXCL10 receptor |
|  | chemokine (C-X-C) receptor 3 |
|  | RP23-49C8.4 |
|  | CXCR3 |
|  | CXCL10 receptor |
|  | G protein-coupled receptor 9 |
|  | IP10 receptor |
|  | OTTHUMP00000070257 |
|  | C-X-C motif chemokine receptor 3 |
|  | IP-10/CXCL10 receptors |
|  | chemokine (C-X-C motif) receptor 3 |
|  | IP10 receptors |
|  | Mig receptors |
|  | chemokine (C-X-C motif) receptor III |
|  | antigen CD183 |
|  | chemokine receptor 3 |
|  | IP10-Rs |
|  | OTTMUSP00000019734 |
|  | IP10-R |
|  | CD182 |
|  | IP-10 receptor |
|  | CXCR-3 |

---

|  |  |
| --- | --- |
| GO ID | 0019957 |
|  | 0016493 |
|  | 0016494 |
|  | 0019956 |
|  | 0004950 |
|  | 0007186 |
|  | 0010818 |
|  | 0001525 |
|  | 0006915 |
|  | 0019722 |
|  | 0007155 |
|  | 0060326 |
|  | 0007166 |
|  | 0070098 |
|  | 0006935 |
|  | 0006955 |
|  | 0006954 |
|  | 0007204 |
|  | 0030155 |
|  | 0002685 |
|  | 0005737 |
|  | 0009897 |
|  | 0005887 |
|  | 0005886 |
|  | 0019958 |
|  | 0004930 |
|  | 0038023 |
|  | 0007189 |
|  | 0071954 |
|  | 0016525 |
|  | 0001937 |
|  | 1900118 |
|  | 0045766 |
|  | 0008284 |
|  | 0050921 |
|  | 1900119 |
|  | 0051281 |
|  | 0045944 |
|  | 0007165 |
|  | 0009986 |
|  | 0016021 |
|  | 0016020 |
|  | 0004872 |
|  | 0006928 |
|  | 0030816 |
|  | 0043950 |
|  | 0005829 |
|  | 0004871 |
|  | 0005622 |
|  | 0019233 |

---

|  |  |
| --- | --- |
| KEGG ID | hsa:2833 |
|  | mmu:12766 |
|  | rno:84475 |

---

|  |  |
| --- | --- |
| Organism | Homo sapiens {Organism urn:agi-taxid:9606} |
|  | Mus musculus {Organism urn:agi-taxid:10090} |
|  | Rattus norvegicus {Organism urn:agi-taxid:10116} |
|  | Homo sapiens |
|  | Mus musculus |
|  | Rattus norvegicus |

---

|  |  |
| --- | --- |
| Mouse chromosome position | X 44.58 cM |
|  | X 41.0 cM |

---

|  |  |
| --- | --- |
| OMIM ID | 300574 |

---

|  |  |
| --- | --- |
| Rat chromosome position | Xq22 |
|  | Xq31 |

---

|  |  |
| --- | --- |
| Hugo ID | 4540 |
|  | HGNC:4540 |

---

|  |  |
| --- | --- |
| Human chromosome position | Xq13.1 |
|  | Xq13 |

---

|  |  |
| --- | --- |
| Swiss-Prot Accession | P49682 |
|  | P49682.2 |
|  | O88410 |
|  | O88410.2 |
|  | Q9JII9 |
|  | Q9JII9.1 |
|  | B2R982 |
|  | O15185 |
|  | Q7Z710 |
|  | Q9P2T4 |
|  | Q9P2T5 |
|  | Q4V9Y6 |
|  | Q9QWN6 |
|  | A2ALY0 |

---

|  |  |
| --- | --- |
| PIR ID | JE0349 |

---

|  |  |
| --- | --- |
| GenBank ID | NC\_000023 |
|  | XM\_017029436 |
|  | XP\_016884925 |
|  | XM\_005262257 |
|  | XP\_005262314 |
|  | XM\_005262256 |
|  | XP\_005262313 |
|  | XM\_017029435 |
|  | XP\_016884924 |
|  | NM\_001504 |
|  | NP\_001495 |
|  | NM\_001142797 |
|  | NP\_001136269 |
|  | NG\_029076 |
|  | AB032735 |
|  | BAA92297 |
|  | AB032736 |
|  | BAA92298 |
|  | AB032737 |
|  | BAA92299 |
|  | AB032738 |
|  | BAA92300 |
|  | AL590763 |
|  | CH471132 |
|  | EAX05283 |
|  | CQ834242 |
|  | CAH05336 |
|  | HI574346 |
|  | CBX47662 |
|  | U32674 |
|  | AAC50505 |
|  | Z79783 |
|  | CAB02143 |
|  | AF469635 |
|  | AAP55851 |
|  | AK313679 |
|  | BAG36429 |
|  | AY242128 |
|  | AAO92295 |
|  | BC034403 |
|  | AAH34403 |
|  | KU178101 |
|  | ALQ33559 |
|  | KU178102 |
|  | ALQ33560 |
|  | X95876 |
|  | CAA65126 |
|  | P49682 |
|  | NC\_000086 |
|  | NM\_009910 |
|  | NP\_034040 |
|  | AL805980 |
|  | CH466564 |
|  | EDL14135 |
|  | AB003174 |
|  | BAA34045 |
|  | AF045146 |
|  | AAC40163 |
|  | BC096626 |
|  | AAH96626 |
|  | O88410 |
|  | NC\_005120 |
|  | NM\_053415 |
|  | NP\_445867 |
|  | AC\_000089 |
|  | AABR07039203 |
|  | AAHX01111638 |
|  | CH473966 |
|  | EDL95884 |
|  | AF223642 |
|  | AAF76982 |
|  | BC091136 |
|  | AAH91136 |
|  | Q9JII9 |
|  | NC\_018934 |
|  | AMYH02039677 |
|  | DQ892460 |
|  | ABM83386 |
|  | DQ895673 |
|  | ABM86599 |
|  | AC\_000042 |
|  | AAHY01215118 |
|  | AC\_000155 |
|  | ABBA01067767 |
|  | ABBA01067768 |
|  | CAM25746 |
|  | NT\_011669 |
|  | NW\_001842373 |
|  | NW\_927711 |
|  | AC\_000066 |
|  | NT\_039706 |
|  | NW\_001035176 |
|  | NW\_001091918 |
|  | NW\_048043 |

---

|  |  |
| --- | --- |
| Swiss-Prot ID | CXCR3\_HUMAN |
|  | CXCR3\_MOUSE |
|  | CXCR3\_RAT |
|  | A2ALY0\_MOUSE |

---

|  |  |
| --- | --- |
| Cell Localization | Cell membrane |
|  | Plasma membrane |

---

|  |  |
| --- | --- |
| Ensembl ID | ENSG00000186810 |
|  | ENSP00000362795.4 |
|  | ENST00000373691.4 |
|  | ENSP00000362797.3 |
|  | ENST00000373693.4 |
|  | ENSMUSG00000050232 |
|  | ENSMUSP00000052444.6 |
|  | ENSMUST00000056614.6 |
|  | ENSRNOG00000003305 |
|  | ENSRNOP00000004406.3 |
|  | ENSRNOT00000004406.5 |
|  | ENSP00000362795 |
|  | ENST00000373691 |
|  | ENSP00000362797 |
|  | ENST00000373693 |
|  | ENSMUSP00000052444 |
|  | ENSMUST00000056614 |
|  | ENSRNOP00000004406 |
|  | ENSRNOT00000004406 |

---

|  |  |
| --- | --- |
| MGI ID | MGI:1277207 |
|  | 1277207 |

---

|  |  |
| --- | --- |
| RGD ID | 621528 |

---

|  |  |
| --- | --- |
| Unigene ID | Hs.198252 |
|  | Mm.12876 |
|  | Rn.24787 |

---

|  |  |
| --- | --- |
| Homologene ID | 1153 |

---

|  |  |
| --- | --- |
| Shape | Stick-vertex |

---

|  |  |
| --- | --- |
| IPI ID | IPI00027754 |
|  | IPI00640020 |
|  | IPI00651732 |
|  | IPI00200582 |
|  | IPI00314938 |

---
